# Evaluating the Effect of Imidacloprid Administered in Artificial Diet on Feeding Behavior of *Diaphorina citri* (Hemiptera: Liviidae) Using Electropenetrography

**DOI:** 10.1093/jee/toy400

**Published:** 2019-01-02

**Authors:** K W Langdon, T A Ebert, M E Rogers

**Affiliations:** 1 Syngenta Crop Protection, Greensboro, NC; 2 Department of Entomology and Nematology, Citrus Research and Education Center, University of Florida, Lake Alfred, FL

**Keywords:** electrical penetration graph, EPG, neonicotinoid, citrus, Asian citrus psyllid

## Abstract

The Asian citrus psyllid, *Diaphorina citri* Kuwayama (Hemiptera: Liviidae) is the vector of *Candidatus* Liberibacter asiaticus (*C*Las), the presumed cause of Huanglongbing (HLB) in citrus. Management strategies were developed in Florida that used soil-applied neonicotinoids to protect young trees. Despite the implementation of intense management programs, infection spread among the most intensively managed groves. We used electopenetrography to test five imidacloprid doses (0.55, 5.5, 55, 550, and 5,500 ppm) administered in artificial diet to approximate the dosage required to reduce feeding activity and prevent salivation/ingestion activity. We failed to detect a significant effect of 0.55 ppm imidacloprid on probing behavior, pathway, or salivation/ingestion activity when compared with the untreated control. We observed a significant reduction in the number of probes and the number of pathway with both 5.5 and 55 ppm imidacloprid. We detected a significant reduction in the number of salivation/ingestion events at both 5.5 ppm and 55 ppm imidacloprid (57 and 54 percent, respectively) compared with the untreated control, and a reduction in number of sustained (>600 s) salivation/ingestion at 55 ppm. While reductions in feeding activity were apparent at dosages of at least 5.5 ppm, we were unable to prevent salivation/ingestion with dosages as high as 5,500 ppm, which is greater than what is known to occur following application in the field. While soil-applied imidacloprid may slow the spread of *C*Las, our findings suggest that prevention of *C*Las inoculation in the field is unlikely. Management strategies must be refined to prevent the spread of HLB in Florida.

The Asian citrus psyllid, *Diaphorina citri* Kuwayama (Hemiptera: Liviidae), was first detected in Florida in 1998 (Halbert and Manjunath 2004) and is known to transmit the phloem-limited proteobacterium, *Candidatus* Liberibacter asiaticus (*C*Las), the presumed cause of citrus greening disease, or Huanglongbing (HLB) (Halbert and Manjunath 2004, Bové 2006, Grafton-Cardwell et al. 2013). Huanglongbing was discovered in Florida in 2005 (Halbert 2005) and has since caused a significant decline in the state’s citrus production (Hodges and Spreen 2015). Upon inoculation of *C*Las into plant phloem, the bacteria move downward into the roots where the root system is severely compromised (Trivedi et al. 2012). Consequently, the canopy is starved of vital nutrients resulting in dead limbs and leaf drop, reductions in fruit yield and quality, with eventual tree death (Halbert and Manjunath 2004, Bové 2006, Grafton-Cardwell et al. 2013). Following the discovery of HLB in Florida citrus, management strategies were quickly developed and focussed on tree health and vector management to aid in reducing the spread of the disease (Rogers 2008). Despite the implementation of intense management programs, virtually all *D. citri* are currently infected with *C*Las, and tree infection continues to spread among the most intensively managed groves (Rogers 2013, Coy and Stelinski 2015). We must evaluate current vector management practices to elucidate why spread of the pathogen continues in order to develop and deliver improved management tactics to growers.

*Diaphorina citri* are characterized as insects with high fecundity and rapid development, undergoing completion of the egg to adult life cycle in as little as 15 d during periods of optimal environmental conditions (Liu and Tsai 2000, Grafton-Cardwell et al. 2013). Adult *D. citri* are attracted to volatiles emitted by newly formed flush shoots where they lay up to 800 eggs per female (Patt and Setamou 2010). If egg lay occurs on *C*Las-infected host tissue, newly hatched nymphs feed on phloem sap and acquire *C*Las (Pelz-Stelinski et al. 2010). Acquisition efficiency is increased for nymphs developing on infected host tissue compared with *D. citri* acquiring the pathogen in the adult stage (Pelz-Stelinski et al. 2010). The resultant dispersal of infected adults causes a spread of the pathogen within and among groves.

Much of *C*Las vector management has maintained focus on young tree programs (Rogers 2008,^2013^). The key objective of the young tree management program is to maintain HLB-free trees until trees reach fruit-bearing age. Young trees flush asynchronously and frequently relative to mature trees in Florida (Hall and Albrigo 2007, Rogers 2012). Because adult *D. citri* seek young flush for egg lay or feeding, young trees are presumably at greatest risk of acquiring *C*Las (Stansly and Rogers 2006). Vector management programs in young trees advise an approximate 3wk alternation between soil-applied neonicotinoids and non-neonicotinoid foliar sprays aimed to maintain *D. citri* populations at low levels in young tree groves (Rogers 2012, Rogers et al. 2014). Neonicotinoids are a unique group of systemic insecticides that when applied to the soil are absorbed by the roots and transported through xylem vascular bundles to the foliage (Elbert et al. 2008). According to the Insecticide Resistance Action Committee (IRAC) neonicotinoids are within the insecticide subgroup 4A, and bind to the insect nicotinic acetylcholine receptor (nAChR) resulting in hyper-excitation, paralysis, and eventual death (IRAC 2017). Three neonicotinoid insecticides are currently labeled for use in nonbearing citrus in Florida: thiamethoxam (Platinum 75 SG—Syngenta Crop Protection, Inc., Greensboro, NC); imidacloprid (Admire Pro 4.6F—Bayer CropScience, Research Triangle Park, NC); and clothianidin (Belay 2.13 SC—Valent USA Corporation, Walnut Creek, CA). A number of studies have investigated the residual activity of neonicotinoids applied to the soil and reported between 6 and 11 wk control (Qureshi and Stansly 2007,^2009^; Ichinose et al. 2010; Setamou et al. 2010; Byrne et al. 2012; Rogers 2012). While factors such as tree size and application rate affect acute neonicotinoid leaf tissue residues (Langdon et al. 2018a), uneven insecticide distribution within a plant is likely to result in areas of sublethal concentrations within leaf tissue at any time following application to the soil (Boina et al. 2009, Rogers 2012).

Electropenetrography (EPG) is the only real-time method used to study and quantify specific feeding behaviors of piercing-sucking hemipterans (Janssen et al. 1989, Joost et al. 2006, Bonani et al. 2010, Cid and Fereres 2010, Butler et al. 2012, Jacobson and Kennedy 2014, Lucini et al. 2016) and rasping-sucking Thysanoptera (Joost and Riley 2005). An EPG monitor is used to identify specific waveforms associated with distinct feeding behaviors. Bonani et al. (2010) correlated repetitive waveforms for *D. citri* with six feeding behaviors including nonprobing (NP), pathway (C), xylem ingestion (G), phloem contact (D), phloem salivation (E1), and phloem ingestion (E2). Occurrence, frequency, and duration of specific waveforms can be used to study insect feeding behavior in response to various stimuli. For example, *D. citri* phloem feeding activities E1 and E2 have been significantly reduced through the use of soil-applied imidacloprid in citrus; however, neither salivation nor ingestion has been prevented to date, and the dosage of imidacloprid received was unknown (Serikawa et al. 2012, Miranda et al. 2016). Understanding the response of particular feeding behaviors, such as salivation or ingestion, can have major implications in pathogen transmission. Coy and Stelinski (2015) speculated that between 80 and 100% of *D. citri* in Florida are infected with *C*Las. Because not all groves are adequately managed for the vector, particularly mature groves and abandoned groves, preventing the inoculation component of the transmission cycle is key to averting the spread of the deadly disease.

Pathogen transmission is fundamentally a two component phenomenon: 1) Acquisition and 2) Inoculation. A number of EPG studies reported a focus of feeding behaviors associated with phloem ingestion (E2) activity as related to *C*Las acquisition (Bonani et al. 2010, Serikawa et al. 2012, Luo et al. 2015, Miranda et al. 2016). Bonani et al. (2010) determined that *D. citri* were able to acquire *C*Las when ingestion behavior (E2) was sustained for 1 h, albeit acquisition success was low (ca. 6%). In contrast, Luo et al. (2015) demonstrated nearly 96% successful *C*Las acquisition by adult *D. citri* with a phloem ingestion (E2) period of as little as 2 min. Moreover, Serikawa et al. (2012) found that *D. citri* were able to perform phloem ingestion (E2) for more than 1 h on citrus tissue containing assumed lethal levels of imidacloprid, yet Miranda et al. (2016) determined that both thiamethoxam and imidacloprid disrupted probing behaviors related to phloem ingestion. Each of the aforementioned studies and resultant conclusions maintained focus on the acquisition/ingestion component of the transmission cycle. While a reduction in acquisition (and subsequent inoculation) of *C*Las is likely to reduce the spread of HLB and could be helpful to the industry, given that citrus is a perennial crop where cumulative effects of disease spread are compounded annually, a simple ‘reduction’ in the spread of *C*Las may no longer be economically viable. Moreover, many groves have become abandoned over recent years throughout Florida, and that space serves as an unmanaged source of inoculum to neighboring groves that remain in production. Consequently, defining the neonicotinoid dose required to deter or prevent salivation into the phloem as related to inoculation is more critical today than the neonicotinoid dose required to reduce or deter ingestion activity (bacterial acquisition) as studied in the past.

The two investigations discussed earlier used EPG to study feeding behavior in response to imidacloprid exposure (Butler et al. 2012, Serikawa et al. 2012, Miranda et al. 2016). These studies each have a single key limitation: imidacloprid dosages to which *D. citri* were exposed are unknown. In both Serikawa et al. (2012) and Miranda et al. (2016), various rates of Admire Pro 4.6F ranging from 0.25 to 0.35 g per plant were applied to the soil of varying plant sizes up to 80 cm tall. While the amount of imidacloprid applied to the soil is known, plant size and physiological activity can both have a significant impact on uptake (Langdon et al. 2018a). Moreover, expression in leaf tissue can only be quantified after the EPG monitoring period using analytical methods such as enzyme-linked immunosorbent assay (ELISA) (Castle et al. 2005, Garlapati 2009, Setamou et al. 2010) or liquid chromatography mass spectrometry (LC–MS) (Langdon 2017). One must chemically analyze the leaf tissue following each EPG monitoring period to develop a mean imidacloprid titer across the test leaf, which likely would not accurately emulate the imidacloprid concentration within the phloem due to potential in-leaf concentration gradients as proposed by Boina et al. (2009), as well as potential changes in concentration during the EPG monitoring period. Because phloem feeding activity is of most interest to researchers studying transmission of *C*Las, knowing the concentration of imidacloprid expressed specifically within the phloem sap is paramount to behavioral studies regarding the *C*Las-*D. citri* transmission matrix.

Despite demonstrations of changes in feeding behavior under the influence of imidacloprid, the imidacloprid dosage required to elicit a particular behavioral response remains unknown (Serikawa et al. 2012, Miranda et al. 2016). The ability to study feeding behavior during ingestion of a range of known imidacloprid dosages would allow us to develop an improved understanding of the effects of imidacloprid exposure to *D. citri* feeding behavior. Unlike EPG studies that used whole plants to study feeding behavior (Serikawa et al. 2012, Miranda et al. 2016), a number of EPG studies used artificial media to evaluate feeding behavior of insects (Joost et al. 2006, Jin et al. 2012, Trebicki et al. 2012). Herein, we describe the first formal study to use EPG to monitor the feeding behavior of *D. citri* during exposure to a sucrose-based liquid diet spiked with five known concentrations of imidacloprid. The overarching goal of this research was to determine the concentration of imidacloprid in citrus leaf tissue required to reduce feeding activity and the concentration required to prevent salivation/ingestion. Ascertaining the imidacloprid concentration required to deter or prevent *D. citri* salivation/ingestion in phloem will allow us to refine current vector management programs which will help either maximize the reduction or perhaps prevent the spread of *C*Las in Florida citrus.

## Materials and Methods

### EPG Assays

Three EPG experiments were conducted to determine the imidacloprid dosage required to reduce feeding activity and prevent salivation/ingestion feeding behaviors when exposed via ingestion. Five imidacloprid dosages were administered across three experiments using a combination of Admire Pro 4.6F and a 30% sucrose-based artificial diet described in detail within Langdon and Rogers (2017). Dosages administered increased with each experiment in attempt to find a dose that prevented salivation altogether, regardless of expected titer in citrus tissue following application to the soil. The first experiment tested 0.55 ppm imidacloprid against an untreated control (*n* = 27, 28, respectively), the second experiment tested 5.5 ppm and 55 ppm imidacloprid against an untreated control (*n* = 27, 31, 26, respectively), and the third experiment tested 550 ppm and 5500 ppm imidacloprid against an untreated control (*n* = 22, 24, 28, respectively). Insects were taken from a colony arbitrarily and could be of any age or sex. No effect of sex on probing behavior has been observed with sample sizes even twice those reported here (T. A. Ebert, personal observations).

**Fig. 1.**
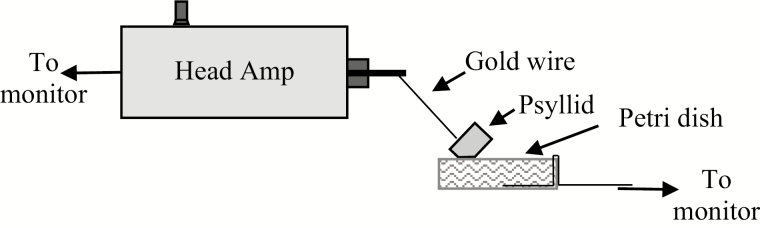
A diagram of the set-up for monitoring *D. citri* on artificial diet.

To monitor insect feeding behavior, the sucrose-based diet, with or without insecticide, was used to fill a polystyrene petri dish 3.5 cm in diameter by 1 cm deep (Corning Glass Works, Corning NY 14831, part #25050-35) (Fig. 1). A 26 AWG copper wire was inserted into the diet, with the tag end folded over the outer rim of the petri dish. Parafilm M (Pechiney Plastic Packaging, Menasha WI 54952) was then stretched over the diet filled petri dish in a manner that prevented air gaps between the undersurface of stretched Parafilm M and top concave surface of liquid diet. The equipment and its set-up were described in detail elsewhere (Ebert and Rogers 2016). In brief, two 4-channel AC-DC monitors (EPG Technologies, Inc., Gainesville, FL) were used in DC mode with 150 mV substrate voltage. Data was acquired through a DI710 AD converter (Akron, OH) using Windaq software at a sampling rate of 100 Hz/channel. *Diaphorina citri* adults were tethered using a 2-cm long by 25.4 µm diameter gold wire (Sigma Cohn Corp., Vernon, NY) attached to the thoracic tergites using silver glue (1:1:1 w:w:w, white glue:water:silver flake [8–10 µm, Inframat Advanced Materials, Manchester CT]). The opposite end of the gold wire was connected to the unit head amp set to an impedance of 10^9^ Ω, and the copper wire from the petri dish was connected to the ‘soil probe’ electrode from the monitor.

**Fig. 2.**
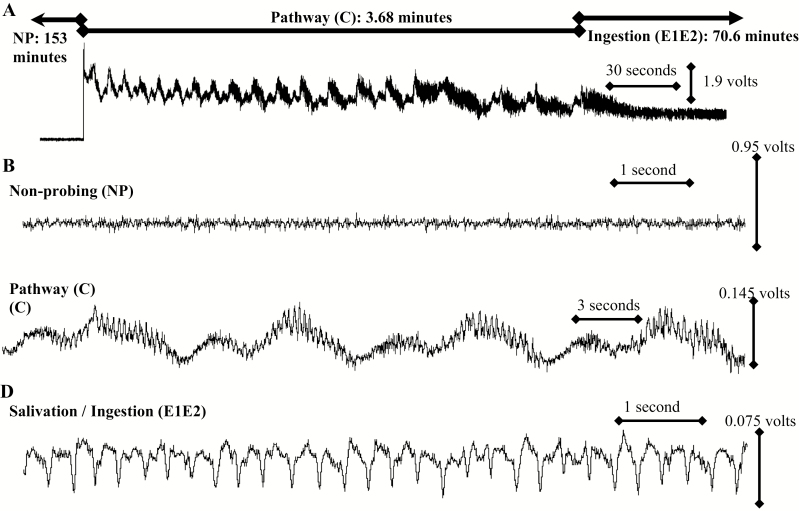
A diagram of EPG waveforms of *D. citri* on artificial diet. (A) Overview of the three EPG waveforms by *D. citri* on artificial diet. (B) Detailed view of nonprobing (NP). (C) Detailed view of pathway (C). (D) Detailed view of salivation/ingestion (E1E2).

Test insects were subjected to a starvation period of 30 min from the time the insects were removed from the colony until they were placed on diet. All insects were wired during this period without being chilled or anesthetized with CO_2_. Recording began before *D. citri* were placed on the Parafilm M covered petri dish to ensure that all recordings started in the NP behavior and recordings were made over a 23 h period. Recording time must be matched to insect biology, and in some cases a 23 h recording time is insufficient (George et al. 2018). The insects, diet, and head amp were contained in a Faraday cage to minimize electronic noise. Light was provided by overhead fluorescent lights [24:0 (L:D) h] and room temperature was maintained at 26.6^o^C. When exposed to plant tissue, *D. citri* are known to exhibit at least six waveforms: nonprobing (NP), pathway (C), phloem contact (D), phloem salivation (E1), phloem ingestion (E2), and xylem ingestion (G) (Bonani et al. 2010). When exposed to artificial diet in this study, three waveforms were identified: nonprobing (NP), pathway (C), and salivation/ingestion (E1E2) (Fig. 2).

With two 4-channel monitors, a three-treatment experiment does not fit evenly. For each run of any given experiment, all treatments occurred at least once. Multiple replicates of the same treatment within a single run were evenly split between two monitors. The position in the room for any one treatment was rotated between runs to ensure that any potential room effects were evenly distributed between all treatments.

### Insect Culture

A continuous culture of laboratory susceptible (LS) *D. citri* was reared at the University of Florida Citrus Research and Education Center in Lake Alfred on *Murraya koenigii* (L.) Spreng. (Sapinales, Rutaceae) maintained at 27°C with RH 65% with a photoperiod of 14:10 (L:D) h. Following establishment in 2005, the LS strain did not receive any exposure to insecticides and routine quantitative real time (qPCR) testing as described in Pelz-Stelinski et al. (2010) was used to confirm the colony was *C*Las-free.

### Plant Uptake

To estimate the maximum level of imidacloprid in plant tissues, 25 citrus (v. Valencia/r.s. Kuharsky) trees were grown in 3.8-liter pots. Each plant was treated with 1 ml of Admire Pro 4.6F in 100 ml water twice per week until symptoms of phytotoxicity were apparent, but plants had not yet started to abort leaves. Plant stems of all ages were cut into 1 cm sections and the bark separated from the wood. The pieces were placed in centrifuge tubes and the phloem and xylem extracted (Hijaz and Killiny 2014). The resulting fluids were analyzed using Ultra-High Performance Liquid Chromatography with a C-18 column coupled to a Thermo TSQ Quantum mass spectrometer (UHPLC-MS) (Langdon et al. 2018a).

### Statistical Analysis

Data analysis used an adaptation of Ebert 2.01 (Ebert and Rogers 2016) that was simplified to deal with a psyllid exhibiting only three waveforms (nonprobing (NP), pathway (C), and salivation/ingestion (E1E2)). There was no clear separation between salivation (E1) and ingestion (E2), therefore, all salivation and ingestion behaviors were pooled into one unit: salivation/ingestion (E1E2). Count data were square root transformed, duration data were log_e_ transformed, and percentage data were logit transformed prior to analysis. Analyses were performed using Proc Glimmix in SAS 9.4M4 running under SAS Enterprise Guide 7.13 (SAS Institute 2013). From these three waveforms 20 variables were calculated for each insect (Table 1). These variable names are used throughout but note that the reported means are across insect. Technically, ‘number of probes’ by insect becomes ‘mean number of probes per insect’ after statistical analysis and this value is no longer a count but an average of counts from multiple insects.

**Table 1.** Description of adult *D. citri* feeding behavior by EPG model abbreviation

Behavior	Abbreviation^*a*^	Behavior description
Probing	NumPrbs	Total number of probing events
	MnPrbs	Mean duration (s) of probing events
	DurFrstPrb	Duration (s) of first probe
Nonprobing	NumNP	Total number of nonprobing events
(NP)	TtlDurNP	Sum of duration (s) of all nonprobing events
	MnDurNP	Mean duration (s) of all nonprobing events
	DurNpFllwFrstSusE1E2	Duration (s) of nonprobing event before first sustained (>600 s) ingestion
Pathway	NmbrC	Number of pathway events
(C)	TtlDurC	Total duration (s) of pathway events
	MnDurC	Mean duration (s) of pathway events
	PrcntPrbC	Percent of probe duration in C
Salivation/Ingestion^*b*^	NumE1E2	Number of salivation/ingestion events
(E1E2)	NumLngE1E2	Number of long (>600 s) salivation/ingestion events
	TtlDurE1E2	Total duration (s) of salivation/ingestion
	MnDurE1E2	Mean duration (s) of salivation/ingestion
	TmFrstSusE1E2StrtPrb	Time (s) until first sustained (>600 s) salivation/ingestion from start of probe with the sustained event
	TmFrstE1E2FrmPrbStrt	Duration (s) of first salivation/ingestion event from start of probe
	PrcntPrbE1E2	Percent of probe duration in salivation/ingestion
	PrcntE1E2SusE1E2	Percent of salivation/ingestion duration spent in sustained (>600 s) salivation/ingestion
	TmFrstSusE1E2	Time (s) to first sustained E1E2 from start of recording

^*a*^All variables are by insect. When used in statistical analyses the resultant means are per insect.

^*b*^There is no clear separation between E1 and E2 in the artificial diet. The waveforms blend one into the other, and separating them would introduce considerable error into the measurements.

## Results and Discussion

As this is the first time that artificial diet was used for *D. citri* in conjunction with EPG, we show an overview of the three waveforms (Fig. 2A). The nonprobing waveform in this view shows background noise (Fig. 2B), though in some cases it also shows behaviors like walking or resting (Youn et al. 2011). The pathway waveform (Fig. 2C) shows the characteristic pattern found in plants and was previously described (Bonani et al. 2010), while the waveform shown in Fig. 2D illustrates salivation/ingestion. We analyzed the frequency of the waveforms (data not presented); however, like observed in previous research, the overlap in frequency of the different waveforms for psyllids makes identification based solely on signal frequency difficult (Bonani et al. 2010, Cid and Fereres 2010).

**Fig. 3.**
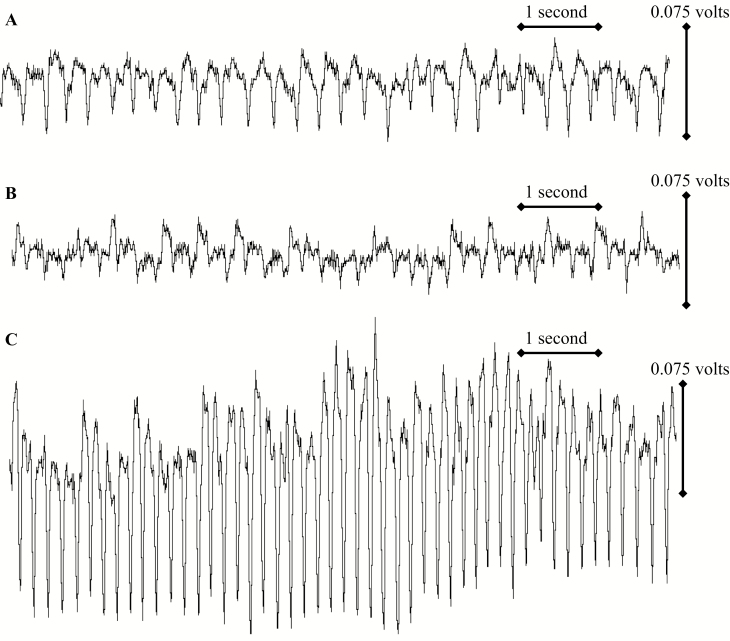
Three variants of the salivation/ingestion (E1E2) waveform from the same insect of *D. citri* feeding on artificial diet.

Characterization of salivation and/or ingestion waveforms under exposure to artificial diet presents a challenge for hemipterous insects when compared with when plant tissue is used. One potential explanation is that insects determine ingestion strategy based on pressure. Phloem is under pressure (Turgeon 2010), while xylem is under tension (Zimmermann 2002, Koch et al. 2004). In contrast, the pressure and tension of artificial diet is near zero. There may be some small change in pressure or tension over the 24 h assay period, depending on changes in room temperature, barometric pressure, or as the tap water used to make the diet warms to room temperature. The insect may not receive enough diet if it uses a phloem ingestion behavior, yet it may require little effort if xylem ingestion muscles are used. In contrast, the artificial diet contains sugar levels equal to or greater than citrus phloem (Killiny 2017). The insect will detect this through precibarial chemosensilla (Backus and Mclean 1985). Therefore, the insect may relate artificial diet with phloem. We observed a clear nonprobing waveform, followed by a waveform that reflects pathway from probing on a plant. This may be followed by a long-lasting waveform of variable shape (Fig. 3). The presence of excrement and lack of mortality suggests that this long-lasting waveform is ingestion. However, a more detailed investigation of this waveform exceeds the scope of this research.

It is important to understand the level of insecticide that is typical in the field and the maximum titer possible when investigating pesticide activity using artificial diet. The observed titer is dependent on where one measures within the plant; however, most psyllids prefer the midrib area of an individual leaf where the level of imidacloprid observed was between 295 and 528 ppb (Langdon et al. 2018b). This was measured by grinding up the relevant portion of the leaf. Converting this observed titer into what the insect experiences in the phloem or xylem is problematic. While soil applied imidacloprid is taken up in the xylem, the phloem has a higher concentration (Table 2). As expected, the metabolites of imidacloprid were only found in the phloem. This suggests that imidacloprid is taken up by the xylem and transported to the leaves where transpiration concentrates the imidacloprid in the leaf, whereupon it is picked up by the phloem. Both the level in the xylem and level in the phloem is important because the psyllid is a phloem feeder on new flush but ingests more xylem when on older tissue (Ebert et al. 2018).

**Table 2.** Levels of imidacloprid, 5-OH, and olefin in xylem and phloem of plants treated with soil applied Admire Pro 4.6F

Metabolite	Imidacloprid		5-OH		Olefin	
Source	Xylem	Phloem	Xylem	Phloem	Xylem	Phloem
Average	32.61	51.52	nd	1.07	<0.05	0.49
Standard deviation	12.08	27.04	nd	1.50	<0.05	0.63
Median	30.08	41.71	nd	0.62	<0.05	0.25
Min	17.19	19.67	nd	0.16	<0.05	0.06
Max	70.90	119.80	nd	7.09	<0.05	2.82

Twenty samples of phloem and xylem were collected, however, two samples of xylem were lost in developing the analytic method.

In the present study, we tested a range of five imidacloprid doses across three experiments to approximate the dosage required to: 1) Reduce feeding activity and 2) Prevent salivation/ingestion activity. In the first experiment, all insects had nonprobing and pathway waveforms. The feeding waveform was found in 53.6% of control insects and 55.6% of treated insects. During the first experiment, we failed to detect a significant effect of 0.55 ppm (550 ppb) imidacloprid on *D. citri* probing behavior, pathway, or salivation/ingestion activity when compared with the untreated control (Table 3). These results indicate that a concentration of 0.55 ppm may not deter *D. citri* feeding activity or prevent E1E2, resulting in a failure to interdict bacterial transmission. In the second experiment, all insects had nonprobing and pathway waveforms. The feeding waveform was found in 80.8% of control insects, 55.6% of the insects are 5.5 ppm, and 64.5% of the insects at 55 ppm. Imidacloprid doses 5.5 (5500 ppb) and 55 ppm (55,000 ppb) generally influenced a majority of probing and pathway parameters (Table 4). A significant reduction in the number of probes (NumPrbs) and the number of pathway events (NmbrC) was observed with both 5.5 and 55 ppm imidacloprid compared with the untreated control. Similarly, Miranda et al. (2016) found that significantly fewer probing and pathway events occurred on plants treated with imidacloprid compared with untreated plants at 35 d following insecticide application to the soil, although the precise dosage within the plant tissues or received by the insect was unknown. However, we failed to detect a reduction in the duration of the first (DurFrstPrb) probe event, a reduction in the percentage of probe in pathway (PrcntPrbC), or a reduction in the percentage of probe time spent in E1E2 (PrcntPrbE1E2), which may indicate that *D. citri* adults were unable to detect imidacloprid at concentrations up to 55 ppm. Miranda et al. (2016) hypothesized that *D. citri* were able to detect imidacloprid treated plants only following a short period of phloem sap ingestion (E2), and went on to conclude that imidacloprid likely acts as a feeding deterrent when applied to the soil. In addition, the total duration of nonprobing (TtlDurNP) and mean duration of nonprobing (MnDurNP) was significantly longer at 55 ppm imidacloprid compared with the untreated control, and the total duration of pathway (TtlDurC) was significantly reduced at 5.5 ppm, and further reduced at 55 ppm. Similarly, Butler et al. (2012) reported extended periods of nonprobing activity for the potato psyllid, *Bactericera cockerelli* (Sulc) (Hemiptera: Triozidae) on potato plants treated with imidacloprid.

**Table 3.** LSMeans ± SEM for each behavioral parameter following exposure of adult *D. citri* to artificial diet with and without 0.55 ppm imidacloprid

Behavior	Parameter	Control	0.55 ppm	*P*-value
		LSMeans ± SE^a^	LSMeans ± SE^a^	
Probing/nonprobing	NumPrbs (sqrt)	7.10 ± 0.48	6.22 ± 0.49	0.2034
	MnPrbs (log)	4.70 ± 0.17	5.02 ± 0.17	0.1843
	DurFrstPrb (lgt)	3.94 ± 0.17	3.95 ± 0.18	0.9712
	NumNP (sqrt)	7.19 ± 0.47	6.29 ± 0.48	0.1884
	TtlDurNP (log)	11.25 ± 0.06	11.15 ± 0.06	0.2503
	MnDurNP (log)	7.52 ± 0.17	7.54 ± 0.18	0.9321
	DurNpFllwFrstSusE1E2 (log)	6.92 ± 7.87	8.85 ± 8.03	0.1563
Pathway (C)	NmbrC (sqrt)	7.22 ± 0.49	6.30 ± 0.50	0.1901
	TtlDurC (log)	8.20 ± 0.19	8.13 ± 0.20	0.8112
	MnDurC (log)	4.49 ± 0.09	4.52 ± 0.09	0.7777
	PrcntPrbC (lgt)	1.19 ± 2.30	4.84 ± 2.16	0.2567
Salivation/Ingestion^*b*^ (E1E2)	NumE1E2 (sqrt)	1.06 ± 0.20	0.86 ± 0.21	0.4883
	NumLngE1E2 (sqrt)	0.53 ± 0.14	0.48 ± 0.14	0.8102
	TtlDurE1E2 (log)	7.81 ± 0.49	7.61 ± 0.49	0.7735
	MnDurE1E2 (log)	6.70 ± 0.49	6.85 ± 0.49	0.8294
	TmFrstSusE1E2StrtPrb (log)	5.45 ± 0.32	4.88 ± 0.34	0.2383
	TmFrstE1E2FrmPrbStrt (log)	4.77 ± 0.24	4.71 ± 0.24	0.8415
	PrcntPrbE1E2 (lgt)	-1.19 ± 0.45	-0.59 ± 0.45	0.3547
	PrcntE2SusE1E2 (lgt)	-1.07 ± 0.42	-0.23 ± 0.39	0.1780
	TmFrstSusE1E2 (log)	11.07 ± 0.11	10.93 ± 0.12	0.3993

Units are either square root transformed (sqrt) for counts, logit transformed for percentages (lgt), or log base e (natural log) transformed for durations (log).

^*a*^All variables are by insect. Means are counts, durations, or percentages per insect, where durations are expressed in seconds.

^*b*^There is no clear separation between E1 and E2 in the artificial diet. The waveforms blend one into the other, and separating them would introduce considerable error into the measurements.

**Table 4.** LS Means ± SEM for each behavioral parameter following exposure of adult *D. citri* to artificial diet with 0, 5.5, or 55 ppm imidacloprid

Behavior	Parameter	Control		5.5 ppm		55 ppm		*P*-value
		LSMeans ± SE^*a*^		LSMeans ± SE^*a*^		LSMeans ± SE^*a*^		
Probing/nonprobing	**NumPrbs**	**8.44 ± 0.46**	**a**	**6.32 ± 0.45**	**b**	**5.31 ± 0.42**	**b**	**<0.0001**
	**MnPrbs**	**4.93 ± 0.17**	**a**	**4.81 ± 0.17**	**ab**	**4.28 ± 0.16**	**b**	**0.013**
	DurFrstPrb	3.61 ± 0.16		3.83 ± 0.16		3.49 ± 0.15		0.2901
	**NumNP**	**8.49 ± 0.45**	**a**	**6.40 ± 0.44**	**b**	**5.42 ± 0.42**	**b**	**<0.0001**
	**TtlDurNP**	**11.17 ± 0.04**	**b**	**11.23 ± 0.04**	**ab**	**11.33 ± 0.04**	**a**	**0.014**
	**MnDurNP**	**6.97 ± 0.15**	**b**	**7.66 ± 0.15**	**a**	**8.09 ± 0.14**	**a**	**<0.0001**
	**DurNpFllwFrstSusE1E2**	**5.59 ± 0.40**	**b**	**9.11 ± 0.55**	**a**	**10.38 ± 0.45**	**a**	**<0.0001**
Pathway (C)	**NmbrC**	**8.64 ± 0.46**	**a**	**6.37 ± 0.45**	**b**	**5.39 ± 0.42**	**b**	**<0.0001**
	**TtlDurC**	**8.78 ± 0.16**	**a**	**7.81 ± 0.16**	**b**	**7.23 ± 0.15**	**c**	**<0.0001**
	**MnDurC**	**4.54 ± 0.09**	**a**	**4.26 ± 0.09**	**ab**	**4.01 ± 0.09**	**b**	**0.0003**
	PrcntPrbC	1.94 ± 1.78		3.08 ± 2.04		4.44 ± 1.74		0.6061
Salivation/Ingestion^*b*^ (E1E2)	**NumE1E2**	**1.72 ± 0.20**	**a**	**0.74 ± 0.19**	**b**	**0.79 ± 0.18**	**b**	**0.0006**
	**NumLngE1E2**	**0.75 ± 0.14**	**a**	**0.39 ± 0.14**	**ab**	**0.29 ± 0.13**	**b**	**0.0497**
	TtlDurE1E2	7.06 ± 0.44		7.02 ± 0.52		6.27 ± 0.45		0.404
	MnDurE1E2	5.85 ± 0.38		6.58 ± 0.45		5.94 ± 0.39		0.4243
	TmFrstSusE1E2StrtPrb	4.87 ± 0.25		4.95 ± 0.32		4.46 ± 0.31		0.4881
	TmFrstE1E2FrmPrbStrt	4.68 ± 0.22		4.76 ± 0.26		4.48 ± 0.22		0.6951
	PrcntPrbE1E2	-1.94 ± 0.46		-0.83 ± 0.54		-1.21 ± 0.47		0.2756
	PrcntE1E2SusE1E2	-1.01 ± 0.37		0.22 ± 1.16		-0.80 ± 0.82		0.6098
	TmFrstSusE1E2	10.70 ± 0.19		10.87 ± 0.19		10.97 ± 0.18		0.581

Units are either square root transformed (sqrt) for counts, logit transformed for percentages (lgt), or log base e (natural log) transformed for durations (log). Bold-face variables where significant differences occurred.

^*a*^All variables are by insect. Means are counts, durations, or percentages per insect, where durations are expressed in seconds.

^*b*^There is no clear separation between E1 and E2 in the artificial diet. The waveforms blend one into the other, and separating them would introduce considerable error into the measurements.

We detected an effect of 5.5 and 55 ppm imidacloprid on two E1E2 parameters: 1) the number of E1E2 events (NumE1E2) and 2) the number of sustained (>600 s) E1E2 events (NumLngE1E2) (Table 4). A significant reduction in the number of E1E2 events was observed at both 5.5 ppm and 55 ppm imidacloprid (57 and 54%, respectively) compared with the untreated control. In addition, the number of sustained (>600 s) E1E2 events was significantly reduced (ca. 61%) at only 55 ppm imidacloprid relative to the untreated control. However, we failed to detect a difference between treatments in the total (TtlDurE1E2) or mean (MnDurE1E2) duration of E1E2. These results demonstrate a reduction in feeding activity (i.e., salivation/ingestion), which presumably would equate to a reduction in bacterial acquisition from *C*Las-infected leaf material in the field, yet a number of *D. citri* were able to successfully salivate or ingest imidacloprid-spiked diet at our highest dose of 55 ppm for a period that exceeded 10 min. An inoculation access period of as little as 15 min is known to result in inoculation of *C*Las into uninfected citrus tissues (Capoor et al. 1974, Grafton-Cardwell et al. 2013), therefore, it remains possible that sustained salivation/ingestion activity exhibited in our study may result in inoculation of *C*Las into uninfected tissue. We failed to detect a difference between 0, 5.5, and 55 ppm imidacloprid in the percent of E1E2 events that resulted in sustained (>600 s) E1E2, time to first E1E2 from start of probe (TmFrstE1E2FrmPrbStrt), nor time to first sustained E1E2 from start of probe (TmFrstSusE1E2StrtPrb), indicating that *D. citri* adults that did undergo salivation/ingestion, did not stop feeding due to imidacloprid detection.

When adult *D. citri* were exposed to 550 ppm imidacloprid, only one insect successfully reached salivation/ingestion behavior for 246 s. Likewise, only one insect successfully reached salivation/ingestion when exposed to 5,500 ppm imidacloprid, which lasted a total of 1,414 s. We were unable to analyze data within the 550 and 5,500 ppm imidacloprid treatments, given the limited number of successful feeding events. Nevertheless, these results would indicate that a few *D. citri* adults will be able to successfully salivate at dosages up to 5,500 ppm imidacloprid when exposed through ingestion. In two separate whole plant studies where small potted citrus plants were drenched with some rate of imidacloprid, a reduction in the number of E1 events was observed (Serikawa et al. 2012, Miranda et al. 2016), yet neither manuscript indicated that E1 was prevented. While insecticide did influence feeding behavior in the present study, our highest imidacloprid dose of 5,500 ppm did not prevent all *D. citri* from undergoing E1E2, therefore, inoculation of *C*Las into uninfected leaf material remains possible at imidacloprid levels as high as 5,500 ppm.

Despite intensive *D. citri* management programs that utilize frequent soil applications of neonicotinoid insecticides, groves continue to succumb to *C*Las infection. We revealed a reduction in a number of probing activities, an increase in nonprobing behaviors (NP), a reduction in pathway behaviors (C), and a reduction in salivation/ingestion behaviors (E1E2) under oral exposure of at least 5.5 ppm imidacloprid-spiked artificial diet using EPG. Reductions in feeding activity observed in the present study confirm findings of previous studies (Butler et al. 2012, Serikawa et al. 2012, Miranda et al. 2016), and are likely to cause a reduction in the spread of HLB within and among commercial citrus groves, providing some level of value in the use of neonicotinoids applied to the soil. Langdon and Rogers (2017) found that the LC_90_ of imidacloprid following ingestion ranged from 62.19 ppm in the lab population to as much as 522.58 ppm in a potentially resistant field collected population, indicating that orally administered imidaclopird residues as high as 55 ppm are sublethal. In addition, they found increased activity when imidacloprid was administered through contact (laboratory susceptible population LC_90_ = 0.13 ppm imidacloprid) than by ingestion (laboratory susceptible population LC_90_ = 62.19 ppm imidacloprid). Nevertheless, while we were able to show significant changes in behavior from orally administered doses of imidacloprid as low as 5.5 ppm, doses up to 5500 ppm were insufficient to prevent all psyllids from exhibiting salivation/ingestion feeding behavior. This is especially problematic because imidacloprid titer following soil-application of Admire Pro 4.6F in commercial groves is not known to exceed 2 ppm (Langdon 2017) and, therefore, imidacloprid applied to the soil at legal field rates is not capable of completely preventing *C*Las inoculation, and thereby prevent the spread of HLB in Florida citrus. Future work should investigate imidacloprid residues following foliar application and resulting *D. citri* feeding behaviors at those concentrations in the attempt to find an application methodology for imidacloprid that is more likely to prevent the spread of *C*Las into uninfected citrus trees.

## References

[CIT0001] Backus E. A., and Mclean D. L. 1985 Behavioral evidence that the precibarial sensilla of leafhoppers are chemosensory and function in host discrimination. Entomol. Exp. Appl. 37: 219–228.

[CIT0002] Boina D. R., Onagbola E. O., Salyani M., and Stelinski L. L. 2009 Antifeedant and sublethal effects of imidacloprid on Asian citrus psyllid, *Diaphorina citri*. Pest Manag. Sci. 65: 870–877. 19431217 10.1002/ps.1767

[CIT0003] Bonani J. P., Fereres A., Garzo E., Miranda M. P., Appezzato-Da-Gloria B., and Lopes J. R. S. 2010 Characterization of electrical penetration graphs of the Asian citrus psyllid, *Diaphorina citri*, in sweet orange seedlings. Entomol. Exp. Appl. 134: 35–49.

[CIT0004] Bové J. M 2006 Huanglongbing: a destructive, newly-emerging, century-old disease of citrus. J. Plant Pathol. 88: 7–37.

[CIT0005] Butler C. D., Walker G. P., and Trumble J. T. 2012 Feeding disruption of potato psyllid, *Bactericera cockerelli*, by imidacloprid as measured by electrical penetration graphs. Entomol. Exp. Appl. 142: 247–257.

[CIT0006] Byrne F. J., Urena A. A., Robinson L. J., Krieger R. I., Doccola J., and Morse J. G. 2012 Evaluation of neonicotinoid, organophosphate and avermectin trunk injections for the management of avocado thrips in *California avocado* groves. Pest Manag. Sci. 68: 811–817. 22396314 10.1002/ps.2337

[CIT0007] Capoor S. P., Rao D. G., and Visvanth S. M. 1974 Greening disease of citrus in the Deccan Trap Country and its relationship with the vector, *Diaphorina citri* Kuwayama, pp. 43–49. *In* Weathers L. G. and Cohen M. (eds.), Proc. 6th Conference of the International Organization of Citrus Virologists. University of California, Richmond.

[CIT0008] Castle S. J., Byrne F. J., Bi J. L., and Toscano N. C. 2005 Spatial and temporal distribution of imidacloprid and thiamethoxam in citrus and impact on *Homalodisca coagulata* populations. Pest Manag. Sci. 61: 75–84. 15593076 10.1002/ps.949

[CIT0009] Cid M., and Fereres A. 2010 Characterization of the probing and feeding behavior of Planococcus citri (Hemiptera: Pseudococcidae) on grapevine. Ann. Entomol. Soc. Am. 103: 404–417.

[CIT0010] Coy M. R., and Stelinski L. L. 2015 Great variability in the infection rate of ‘*Candidatus’* Liberibacter asiaticus in field populations of *Diaphorina citri* (Hemiptera: Liviidae) in Florida. Fla. Entomol. 98: 356–357.

[CIT0011] Ebert T. A., and Rogers M. E. 2016 Effect of substrate voltage on EPG recordings of ingestion and probing behavior in *Diaphorina citri* (Hemiptera: Liviidae). Fla. Entomol. 99: 528–534.

[CIT0012] Elbert A., Haas M., Springer B., Thielert W., and Nauen R. 2008 Applied aspects of neonicotinoid uses in crop protection. Pest Manag. Sci. 64: 1099–1105. 18561166 10.1002/ps.1616

[CIT0013] Ebert T. A., Backus E. A., Shugart H. J., and Rogers M. E. 2018 Behavioral plasticity in probing by *Diaphorina citri* (Hemiptera, Liviidae): ingestion from phloem versus xylem is influenced by leaf age and surface. J. Insect Behav. 31: 119–137. 29628621 10.1007/s10905-018-9666-0 PMC5882765

[CIT0014] Garlapati S 2009 Uptake of soil-applied neonicotinoids by citrus plants and their impact on selected biological parameters of the Asian citrus psyllid, Diaphorina citri Kuwayama. M.S. thesis, Texas A&M University-Kingsville, Kingsville.

[CIT0015] George J., Ammar E. D., Hall D. G., Shatters R. G. Jr, and Lapointe S. L. 2018 Prolonged phloem ingestion by *Diaphorina citri* nymphs compared to adults is correlated with increased acquisition of citrus greening pathogen. Sci. Rep. 8: 10352. 29985396 10.1038/s41598-018-28442-6 PMC6037740

[CIT0016] Grafton-Cardwell E. E., Stelinski L. L., and Stansly P. A. 2013 Biology and management of Asian citrus psyllid, vector of the huanglongbing pathogens. Annu. Rev. Entomol. 58: 413–432. 23317046 10.1146/annurev-ento-120811-153542

[CIT0017] Halbert S. E. 2005 The discovery of huanglongbing in Florida. *In* Proceedings of the 2nd International Citrus Canker and Huanglongbing Research Workshop. Florida Citrus Mutual, Orlando, FL 7–11 November 2005.

[CIT0018] Halbert S. E., and Manjunath K. L. 2004 Asian citrus psyllids (Sternorrhyncha: Psyllidae) and greening disease of citrus: a literature review and assessment of risk in Florida. Fla. Entomol. 87: 330–353.

[CIT0019] Hall D. G., and Albrigo L. G. 2007 Estimating the relative abundance of flush shoots in citrus with implications on monitoring insects associated with flush. Hort. Sci. 42: 364–368.

[CIT0020] Hijaz F., and Killiny N. 2014 Collection and chemical composition of phloem sap from *Citrus sinensis* L. Osbeck (Sweet Orange). PLoS One. 9: e101830. 25014027 10.1371/journal.pone.0101830 PMC4094394

[CIT0021] Hodges A. W., and Spreen T. H. 2015 Economic contributions of the Florida citrus industry in 2014–15 and for reduced production, pp. 1–9. University of Florida, Food and Resource Economics Department, Gainesville, FL.

[CIT0022] Ichinose K., Bang D. V., Tuan d. o. H., and Dien l. E. Q. 2010 Effective use of neonicotinoids for protection of citrus seedlings from invasion by *Diaphorina citri* (Hemiptera: Psyllidae). J. Econ. Entomol. 103: 127–135. 20214377 10.1603/ec09218

[CIT0023] Insecticide Resistance Action Committee (IRAC) 2017 IRAC mode of action classification scheme v. 8.2 Crop Life International. 1–26.

[CIT0024] Jacobson A. L., and Kennedy G. G. 2014 Electrical penetration graph studies to investigate the effects of cyantraniliprole on feeding behavior of *Myzus persicae* (Hemiptera: Aphididae) on *Capsicum annuum*. Pest Manag. Sci. 70: 836–840. 23943625 10.1002/ps.3626

[CIT0025] Janssen J., Tjallingii W., and Lenteren J. 1989 Electrical recording and ultrastructure of stylet penetration by the greenhouse whitefly. Entomol. Exp. Appl. 52: 69–81.

[CIT0026] Jin S. Z. M., Chen E. A. Backus, Sun X. L., and Xiao B. 2012 Characterization of EPG waveforms for the tea green leafhopper, *Empoasca vitis* Göthe (Hemiptera: Cicadellidae), on tea plants and their correlation with stylet activities. J. Insect Physiol. 58: 1235–1244. 22750027 10.1016/j.jinsphys.2012.06.008

[CIT0027] Joost P. H., and Riley D. G. 2005 Imidacloprid effects on probing and settling behavior of *Frankliniella fusca* and *Frankliniella occidentalis* (Thysanoptera: Thripidae) in tomato. J. Econ. Entomol. 98: 1622–1629. 16334332 10.1093/jee/98.5.1622

[CIT0028] Joost P. H., Backus E. A., Morgan D., and Yan F. 2006 Correlation of stylet activities by the glassy-winged sharpshooter, *Homalodisca coagulata* (Say), with electrical penetration graph (EPG) waveforms. J. Insect Physiol. 52: 327–337. 16427072 10.1016/j.jinsphys.2005.11.012

[CIT0029] Killiny N 2017 Metabolite signature of the phloem sap of fourteen citrus varieties with different degrees of tolerance to *Candidatus* Liberibacter asiaticus. Physiol. Mol. Plant Pathol. 97: 20–29.

[CIT0030] Koch G. W., Sillett S. C., Jennings G. M., and Davis S. D. 2004 The limits to tree height. Nature. 428: 851–854. 15103376 10.1038/nature02417

[CIT0031] Langdon K. W 2017 Optimizing the use of soil-applied neonicotinoids for control of Diaphorina citri Kuwayama (Hemiptera: Psyllidae) in young citrus trees. Ph.D. dissertation, University of Florida.

[CIT0032] Langdon K. W., and Rogers M. E. 2017 Neonicotinoid-induced mortality of *Diaphorina Citri* (Hemiptera: Liviidae) is affected by route of exposure. J. Econ. Entomol. 110: 2229–2234. 28961723 10.1093/jee/tox231

[CIT0033] Langdon K. W., Schumann R., Stelinski L. L., and Rogers M. E. 2018a Influence of tree size and application rate on expression of thiamethoxam in citrus and its efficacy against *Diaphorina citri* (Hemiptera: Liviidae). J. Econ. Entomol. 111: 770–779. 29471401 10.1093/jee/toy001 PMC6019049

[CIT0034] Langdon K. W., Schumann R., Stelinski L. L., and Rogers M. E. 2018b Spatial and temporal distribution of soil-applied neonicotinoids in citrus tree foliage. J. Econ. Entomol. 111: 1788–1798. 10.1093/jee/toy114 PMC6075369 29688422

[CIT0035] Liu Y. H., and Tsai J. H. 2000 Effects of temperature on biology and life table parameters of the Asian citrus psyllid, *Diaphorina citri* Kuwayama (Homoptera: Psyllidae). Ann. Appl. Biol. 137: 201–206.

[CIT0036] Lucini T., Panizzi A. R., and Backus E. A. 2016 Characterization of an EPG waveform library for redbanded stink bug, *Piezodorus guildinii* (Hemiptera: Pentatomidae), on soybean plants. Ann. Entomol. Soc. Am. 109: 198–210.

[CIT0037] Luo X., Yen A. L., Powell K. S., Wu F., Wang Y., Zeng L., Yang Y., and Cen Y. 2015 Feeding behavior of *Diaphorina citri* (Hemiptera: Liviidae) and its acquisition of ‘*Candidatus* Liberibacter asiaticus’, on huanglongbing-infected *Citrus reticulata* leaves of several maturity stages. Fla. Entomol. 98: 186–192.

[CIT0038] Miranda M. P., Yamamoto P. T., Garcia R. B., Lopes J. P., and Lopes J. R. 2016 Thiamethoxam and imidacloprid drench applications on sweet orange nursery trees disrupt the feeding and settling behaviour of *Diaphorina citri* (Hemiptera: Liviidae). Pest Manag. Sci. 72: 1785–1793. 26694803 10.1002/ps.4213

[CIT0039] Patt J. M., and Sétamou M. 2010 Responses of the Asian citrus psyllid to volatiles emitted by the flushing shoots of its rutaceous host plants. Environ. Entomol. 39: 618–624. 20388295 10.1603/EN09216

[CIT0040] Pelz-Stelinski K. S., Brlansky R. H., Ebert T. A., and Rogers M. E. 2010 Transmission parameters for *Candidatus* liberibacter asiaticus by Asian citrus psyllid (Hemiptera: Psyllidae). J. Econ. Entomol. 103: 1531–1541. 21061950 10.1603/EC10123

[CIT0041] Qureshi J. A., and Stansly P. A. 2007 Integrated approaches for managing the Asian citrus psyllid *Diaphorina citri* (Homoptera: Psyllidae) in Florida. Proc. Fla. State Hort. Soc. 120: 110–115.

[CIT0042] Qureshi J. A., and Stansly P. A. 2009 Insecticidal control of Asian citrus psyllid *Diaphorina citri* (Hemiptera: Psyllidae). Proc. Fla. State Hortic. Soc. 122: 172–175.

[CIT0043] Rogers M. E 2008 General pest management considerations. Citr. Indus. 89: 12–17.

[CIT0044] Rogers M. E 2012 Protection of young trees from the Asian citrus psyllid and HLB. Citr. Indus. 93: 10–15.

[CIT0045] Rogers M. E. 2013 Asian citrus psyllid management for young trees. *In* Proceedings, Florida Citrus Growers’ Institute, 2 April 2013, Avon Park, FL.

[CIT0046] Rogers M. E., Stansly P. A., and Stelinski L. L. 2014 2014 Florida citrus pest management guide: Asian citrus psyllid and citrus leafminer. UF/IFAS Extens. ENY-734. University of Florida, Gainesville, FL.

[CIT0047] SAS Institute 2013 SAS/IML User’s Guide, Version 9.4. SAS Institute, Cary, NC.

[CIT0048] Serikawa R. H., Backus E. A., and Rogers M. E. 2012 Effects of soil-applied imidacloprid on Asian citrus psyllid (Hemiptera: Psyllidae) feeding behavior. J. Econ. Entomol. 105: 1492–1502. 23156142 10.1603/EC11211

[CIT0049] Sétamou M., Rodriguez D., Saldana R., Schwarzlose G., Palrang D., and Nelson S. D. 2010 Efficacy and uptake of soil-applied imidacloprid in the control of Asian citrus psyllid and a citrus leafminer, two foliar-feeding citrus pests. J. Econ. Entomol. 103: 1711–1719. 21061971 10.1603/ec09371

[CIT0050] Stansly P. A., and Rogers M. E. 2006 Managing Asian citrus psyllid populations. Citr. Indus. 87: 17–19.

[CIT0051] Trebicki P., Tjallingii W. F., Harding R. M., Rodoni B. C., and Powell K. S. 2012 EPG monitoring of the probing behaviour of the common brown leafhopper *Orosius orientalis* on artificial diet and selected host plants. Arthropod-Plant Interact. 6: 405–415.

[CIT0052] Trivedi P., He Z., Van Nostrand J. D., Albrigo G., Zhou J., and Wang N. 2012 Huanglongbing alters the structure and functional diversity of microbial communities associated with citrus rhizosphere. Isme J. 6: 363–383. 21796220 10.1038/ismej.2011.100 PMC3260493

[CIT0053] Turgeon R 2010 The puzzle of phloem pressure. Plant Physiol. 154: 578–581. 20921188 10.1104/pp.110.161679 PMC2949042

[CIT0054] Youn Y., Backus E. A., Serikawa R. H., and Stelinski L. L. 2011 Correlation of an electrical penetration graph waveform with walking by Asian citrus psyllid, *Diaphorina citri* (Hemiptera: Psyllidae). Fla. Entomol. 94: 1084–1087.

[CIT0055] Zimmermann M. H 2002 Xylem structure and the ascent of sap. Springer-Verlag, New York.

